# 1845. Oritavancin Compared to the Standard-of-Care for the Treatment of Gram-positive Blood Stream Infections

**DOI:** 10.1093/ofid/ofac492.1474

**Published:** 2022-12-15

**Authors:** Ryan P Moenster, Ashleigh Wallace-Lacey, Michael Lorenz, Danielle Skouby, Rebecca Fong, Anosha Abdulbasir, Hartley Abner, Justin Alberts, Johnathan Doty, Seth Tiefenauer, Hannah Western, Travis W Linneman

**Affiliations:** St. Louis College of Pharmacy at UHSP/VA St. Louis HCS, St. Louis, Missouri; VA St. Louis HCS, St. Louis, Missouri; VA St. Louis HCS, St. Louis, Missouri; VA St. Louis HCS, St. Louis, Missouri; VA St. Louis HCS, St. Louis, Missouri; St. Louis College of Pharmacy at UHSP, St. Louis, Missouri; St. Louis College of Pharmacy at UHSP, St. Louis, Missouri; St. Louis College of Pharmacy at UHSP, St. Louis, Missouri; St. Louis College of Pharmacy at UHSP, St. Louis, Missouri; St. Louis College of Pharmacy at UHSP, St. Louis, Missouri; St. Louis College of Pharmacy at UHSP, St. Louis, Missouri; VA St. Louis HCS, St. Louis, Missouri

## Abstract

**Background:**

Data is limited comparing oritavancin (ORT) to the standard-of-care (SOC) for the treatment gram-positive blood stream infections (BSI).

**Methods:**

This was a retrospective study of all patients in the Veteran’s Affairs Health Care System treated with at least 1 dose of oritavancin or at least 5 days of vancomycin, daptomycin, ceftaroline, ampicillin, ampicillin-sulbactam, nafcillin, oxacillin, or cefazolin for a documented gram-positive BSI from 1 January 2015 to 30 June 2021. Patients with polymicrobial blood cultures or positive cultures from other sites were included if the organisms were sensitive to the incident antimicrobial; no concomitant antimicrobials could be used once the incident agent was started. Individuals were also excluded if they were diagnosed with endocarditis, had a neutrophil count < 500 cells/mm^3^, or had a diagnosis of Human Immunodeficiency Virus (HIV) on their problem list.

The primary composite outcome was clinical failure, defined as all-cause mortality within 30-days from the end of therapy, or blood cultures positive for the incident organisms ≥72 hours after administration of the first dose and ≤30 days after the administration of the final dose of the study antimicrobial, or any drug or line-related readmissions within 30-days of hospital discharge. Secondary outcomes included all-cause 30-day readmission and rates of acute kidney injury (AKI).

**Results:**

Two hundred-forty patients were identified for screening with 96 meeting criteria (27 in ORT and 69 in SOC groups). Baseline characteristics were generally balanced between groups except more patients in the ORT group received > 96-hours of treatment before the incident antimicrobial was started (70.3% (19/27) vs. 13.04% (9/69); *p<*0.001). The pathogen most prevalent was methicillin-susceptible *Staphylococcus aureus* (MSSA) (ORT 33.3% (9/27) vs. SOC 46.4% (32/69)). Clinical failure occurred in 7.4% (2/27) in the ORT group and 17.4% (12/69) in SOC (*p*=0.34). No components of the primary outcome were significantly different between groups, but AKI did occur more commonly in the SOC group (27.5% (19/69) vs. 3.7% (1/27); *p*=0.01).

**Conclusion:**

ORT appears to be a safe and effective option when directly compared to the SOC for non-endocarditis BSIs.

**Disclosures:**

**Ryan P. Moenster, Pharm.D., FIDSA**, Melinta Therapeutics: Grant/Research Support|Melinta Therapeutics: Honoraria.

